# A comparison of the sensitivity of EQ-5D, SF-6D and TTO utility values to changes in vision and perceived visual function in patients with primary open-angle glaucoma

**DOI:** 10.1186/1471-2415-12-43

**Published:** 2012-08-21

**Authors:** Fiammetta Maria Bozzani, Yasmene Alavi, Mireia Jofre-Bonet, Hannah Kuper

**Affiliations:** 1 London School of Hygiene and Tropical Medicine, Keppel Street, WC1E 7HT, London, UK; 2 City University, Economics, London, UK

**Keywords:** Glaucoma, Quality of life, Utility values, Cost-utility analysis

## Abstract

**Background:**

Economic viability of treatments for primary open-angle glaucoma (POAG) should be assessed objectively to prioritise health care interventions. This study aims to identify the methods for eliciting utility values (UVs) most sensitive to differences in visual field and visual functioning in patients with POAG. As a secondary objective, the dimensions of generic health-related and vision-related quality of life most affected by progressive vision loss will be identified.

**Methods:**

A total of 132 POAG patients were recruited. Three sets of utility values (EuroQoL EQ-5D, Short Form SF-6D, Time Trade Off) and a measure of perceived visual functioning from the National Eye Institute Visual Function Questionnaire (VFQ-25) were elicited during face-to-face interviews. The sensitivity of UVs to differences in the binocular visual field, visual acuity and visual functioning measures was analysed using non-parametric statistical methods.

**Results:**

Median utilities were similar across Integrated Visual Field score quartiles for EQ-5D (P = 0.08) whereas SF-6D and Time-Trade-Off UVs significantly decreased (p = 0.01 and p = 0.001, respectively). The VFQ-25 score varied across Integrated Visual Field and binocular visual acuity groups and was associated with all three UVs (P ≤ 0.001); most of its vision-specific sub-scales were associated with the vision markers. The most affected dimension was driving. A relationship with vision markers was found for the physical component of SF-36 and not for any dimension of EQ-5D.

**Conclusions:**

The Time-Trade-Off was more sensitive than EQ-5D and SF-6D to changes in vision and visual functioning associated with glaucoma progression but could not measure quality of life changes in the mildest disease stages.

## Background

Glaucoma is the second leading cause of blindness worldwide, representing 12.3% of the global burden [1]. Primary open-angle glaucoma (POAG) is the most widespread form among Western populations; if untreated, this chronic degenerative optic neuropathy induces progressive and irreversible loss of peripheral visual field (VF) with tunnel vision and, eventually, blindness. POAG prevalence is 2% among adults (>40 yrs) in high-income countries and is predicted to rise with population ageing, in turn expanding demand for therapies [2]. As health care resources become constrained, the importance of evaluating the economic viability of different treatment options is increasing [3].

Interventions should be compared through cost-utility analysis based on Quality-Adjusted Life-Years (QALYs), a common metric of benefit whose components are life expectancy and Utility Values (UVs) [4]. The latter is a preference-based measure of the quality of life (QOL) associated with different health states, ranging between 0 (death) and 1 (perfect health) [3,5]. Accurate estimates of changes in UVs for progressive stages of visual field loss (VFL) are needed to calculate QALYs gained from glaucoma interventions [6]. Elicitation methods should therefore detect genuine changes in QOL from the very early stages of POAG and beyond. These changes are defined as the minimally important difference in score (i.e. the smallest difference perceived by patients as beneficial), which would call for implementing the intervention in the absence of side effects and excessive cost [7].

Different methods for measuring UVs in POAG patients, including both direct and indirect utility elicitation, have been described in the literature, showing various strenghts of association with VFL [6,8-13]. High frequencies of ceiling effects, which can lead to underestimating QOL changes, have been reported for UV instruments among these studies [14]. In addition, the insensitivity of standard UV elicitation methods, such as the EuroQoL EQ-5D advocated by the National Institute for Health and Clinical Excellence for use in economic evaluations, has been criticised among ophthalmic and non-ophthalmic populations [15,16]. To date, few economic evaluations on glaucoma interventions have been conducted, with a recent systematic review calling for more research in light of the scarcity and poor quality of existing economic evidence compared to the wealth of new technologies to assess [17]. Similarly, there have been limited investigations directly comparing the sensitivity of different UV elicitation methods in POAG research [12,18].

As for the aspects of daily living affected by POAG, few studies currently describe stage-by-stage losses in different QOL dimensions [6,8,19]. However, a considerable body of evidence gathered using the National Eye Institute Visual Function Questionnaire (VFQ-25) indicates that QOL decreases from the early stages of visual field deterioration, with the most affected areas being driving and outdoor mobility [6]. Concerns with activities requiring near vision only emerge when field damage is severe enough to affect binocular central acuity [20,21].

Thus, UVs used in cost-utility analysis for POAG should be sensitive to QOL changes associated with disease progression, yet there is limited evidence on which UV elicitation methods are most sensitive to these changes. The study objective is to identify among 3 widely used methods (EQ-5D, Short Form SF-6D, Time-Trade-Off) the one most sensitive to changes in both binocular VFL and visual functioning (measured using the VFQ-25). In particular, the following psychometric properties of the elicitation methods will be assessed: known-group differences (ability to discriminate among patients on different levels of condition severity) and convergent validity (extent to which the UVs correlate with the visual functioning score) [22].

## Methods

This paper is part of a wider study on QOL in glaucoma patients. It follows an article recently published by Alavi et al. (2011), aimed at developing an algorithm to calculate UVs for POAG patients based on a combination of visual acuity (VA), visual field and contrast sensitivity tests [23]. Full details of study methods are published in Alavi et al. (2011). Ethics approval was granted by Moorfields and Whittington Local Research Ethics Committee and the London School of Hygiene & Tropical Medicine Ethics Committee.

### Design and sample

A sample of outpatients with POAG in one or both eyes was recruited from Moorfields Eye Hospital (MEH, London, UK). After identification through clinical records, those who consented to participate undertook questionnaires and visual tests at MEH on the day their next scheduled consultation occurred.

For eligibility, patients had to be at least 18 years old, English-speaking and free from conditions preventing reliable visual testing and interviewing. Exclusion criteria were eye surgery in the preceding 6 weeks and any ocular co-morbidities contributing to loss of vision.

### Clinical measures of visual function

Binocular measures of VF were selected as more relevant to perceived visual ability. Integrated Visual Field (IVF) scores were used to measure binocular VF as they are derived from routine monocular VF threshold tests and have been demonstrated to predict self-reported visual disabilities better than the Esterman test [24]. Monocular Humphrey 24–2 full threshold tests were performed in both eyes (Humphrey Field Analyzer II, model 730; Humphrey Instruments, Dublin, CA, USA). The maximum sensitivity (dB) recorded between the 52 overlapping points of the right and left monocular fields was used to generate a 52-point integrated (binocular) VF. Points were then scored (<10 dB = 2; 10-19 dB = 1; ≥20 dB = 0) and values summed up to obtain individual IVF scores ranging from 0 (>20 dB in all 52 points) to 104 (<10 dB in all 52 points), respectively the best and worst binocular VF [24]. Only scores obtained through a reliable VF test, as defined by published criteria, were included in the analysis [25]. IVF scores were based on the monocular VF for participants with (1) no perception of light (NPL) in one eye, or (2) severe visual loss (mean deviation ≤ −25 dB) in one eye in their most recent test, and whose eyesight had deteriorated to the extent that their VF was unobtainable in that eye. An IVF score of 104 was designated to those participants who had NPL/severe visual loss in both eyes, such that a reliable VF was unobtainable from either eye. Lacking a universally recognised glaucoma staging system, IVF quartiles were used as a measure of VFL severity.

Visual acuity, both monocular and binocular (VA_B_), was assessed under standardised conditions using a back-illuminated ETDRS logMAR chart (Lighthouse International, New York, NY) read with the aid of the participants’ habitual distance glasses at 4 meters, or 2/1 metres if the letters on the top line could not easily be read at 4/2 metres, respectively. Patients unable to see letters at 1 metre were assigned a value of 1.85 logMAR, (counting fingers), or 2.3 logMAR (vision of hand movements or less) [26].

### Interviews and UV/perceived visual function measurement

Questionnaires and visual tests were administered by the same researcher (YA) at MEH. Information was collected on age, glaucoma diagnosis, gender, ethnicity, education, marital status, living arrangements, use of topical medication, previous glaucoma surgical or laser interventions and time since diagnosis. A depression screener was also administered [27]. Socio-economic status was recorded using the occupational-based UK five-class National Statistics Socio-Economic Classification System [28]. A trained counsellor was available to participants upset by any part of the interview.

Utility measures: The present analysis employs 3 elicitation methods. Two are multi-attribute utility classification systems providing preferences associated with generic health states (EQ-5D, SF-6D), while the Time-Trade-Off (TTO) directly elicits preferences associated with current visual state. The EQ-5D consists of five questions on mobility, self-care, usual activities, pain/discomfort and anxiety/depression, that can take one of three responses representing different levels of problems (none/moderate/extreme). Individual sets of answers are scored according to the health state they represent and converted to UVs [29]. The Medical Outcomes Study Short Form Questionnaire (SF-36) contains 36 items assessing 8 domains of daily living on a 0–100 scale, yielding the norm-based physical and mental component summary scores [30,31]. Six of the 8 dimensions can be used to generate UVs (SF-6D) [32]. A two-part TTO question widely used in ophthalmic research with demonstrated validity and test-retest reliability was applied in this study [33-38]. Participants were first asked how many more years they expected to live (Y), and then to quantify how many of those years – if any- they were willing to trade for perfect vision. The UVs were calculated from the maximum number of years that the person was willing to trade (Z) as follows: UV = (Y − Z)/Y. Thus, the method elicits stated (rather than revealed) preferences.

Perceived visual function measure: VFQ-25 comprises 25 questions used to calculate 12 sub-scales (one assessing general health and the remainder targeting vision-specific functioning) and one composite score between 0 and 100 [39]. Lower scores indicate lower quality of life.

### Data analysis

Data were double-entered on an Access-based database, checked using EpiInfo™ Data Compare and analysed with STATA v.11 (StataCorp LP, College Station, TX, USA). As the main vision and QOL measures were not normally distributed, even after log transformation, a preliminary investigation of the statistical association between variables was performed using non-parametric Spearman’s rank correlation coefficient. Differences between the three sets of UVs were tested with the Wilcoxon signed-rank test. Next, the distribution of median UVs and QOL scores across IVF quartiles was analysed graphically. Associations between (1) the vision markers (IVF and VA_B_) and the UVs and (2) the vision markers and the VFQ-25 composite score were tested using the Kruskal-Wallis test, a non-parametric equivalent of ANOVA. Individual socio-demographic and clinical characteristics were considered as potential confounders. As nonparametric tests do not allow to control for confounding, their associations with the vision markers, the UVs and the VFQ-25 score were independently tested. Individual sub-scales from EQ-5D, SF-36 and VFQ-25 were analysed graphically and using Spearman’s correlation coefficients to assess which dimensions of generic and vision-specific QOL were most affected by glaucoma-induced VFL.

## Results

The characteristics of the 132 patients recruited are summarised in Table 1. Most patients (65%) had been diagnosed with glaucoma over 10 years prior to the study and nearly 70% had not undergone surgery. The full range of IVF scores was represented, although their distribution was skewed towards mild/moderate VFL (median = 29, IQR: 7.5 - 58, range: 0 – 104). The IVF quartiles were: 0–7 (Q1), 8–29 (Q2), 30–59 (Q3), 60–104 (Q4). The VA_B_ of most patients was within the normal range (median = 0.1, IQR: 0–0.3, range: -0.18 - 1.85).

**Table 1:** Socio-demographic and clinical characteristics of patients with POAG (n = 132)

	**N**	**Mean (SD) or %**	**Range**
*Socio-demographic*			
Gender			
Female (%)	61	46.2%	
Male (%)	71	53.8%	-
Age, mean (SD)	132	71.8 (11.0)	27.6 - 93.5
Living conditions			
Living alone (%)	49	37.1%	-
Not living alone (%)	83	62.9%	
		
Employment			
Currently employed (%)	27	20.5%	-
Retired (%)	105	79.5%	
*Clinical*			
Years since POAG diagnosis, mean (SD)	128	14.3 (8.9)	0.28 - 40.6
Type of glaucoma*			
High tension	115	87.1%	-
Normal Tension	17	12.9%	
Currently using eye-drops (%)			
Yes	122	92.4%	-
No	10	7.6%	
Failed depression screener (%)			
Yes	10	7.6%	-
No	122	92.4%	
*Visual*			
IVF , mean (SD)**	124	33.6 (37.6)	0 - 104
Better-seeing eye VA (logMAR), mean (SD)	132	0.3 (0.4)	−0.2 - 2.3
VA_B_ (logMAR)_,_ mean (SD)	132	0.2 (0.4)	−0.18 - 1.85
Best-eye mean deviation, mean (SD)	101	−11.5 (8.2)	−29.6 – 1.10
Worse-eye mean deviation, mean (SD)	122	−18.9 (8.2)	−31.9 – 0.4

* High and normal tension glaucoma defined on the basis of intraocular pressure (normal: 10–21 mm Hg; high: >21 mm Hg).

**Higher values represent increasing visual field loss. 8 patients were unable to score a reliable VF test due to low vision (n = 124/132).

IVF = integrated visual field score.

VA_B_ = binocular visual acuity.

None of the variables listed in Table 1 was simultaneously associated with ophthalmic and QOL measures, thus ruling out major confounding and justifying the use of unadjusted values. In particular, UVs did not change with age, *a priori* considered an important potential confounder (Kruskal-Wallis P = 0.20 for EQ-5D; P = 0.08 for SF-6D; P = 0.48 for TTO).

Table 2 provides a summary of VFQ-25 composite scores and UVs derived from EQ-5D, SF-6D and TTO. Their frequency distributions were heavily skewed towards higher values, especially for TTO, whereby 79/123 respondents reported UV = 1. The three questionnaires yielded significantly different UVs (P ≤ 0.001 for all tests), those from TTO being the highest. Less than 1% of all items were missing for both EQ-5D and SF-6D. Utilities could not be assigned to patients who did not answer one or more questions used in the calculation algorithm (n = 6). As for TTO, a common reason for refusal was inability to consider the question independently from religious beliefs (cited in 4/9 cases).

**Table 2:** Summary of utility values and VFQ-25 scores

	**N**	**Mean (SD)**	**Median (IQR)**	**Observed range**	Possible range*
**EQ-5D**	131	0.8 (0.2)	0.8 (0.7 - 1.0)	−0.1 - 1.0	−0.6 – 1.0
**SF-6D**	126	0.7 (0.1)	0.7 (0.6 - 0.9)	0.4 - 0.9	0.3 – 1.0
**TTO**	123	0.9 (0.2)	1.0 (0.8 - 1.0)	0.2 - 1.0	0 – 1.0
**VFQ-25**	132	72.9 (22.1)	81.1 (57.8 - 91.6)	17.1 - 99.4	0 – 100

* Higher values indicate better health.

EQ-5D = UVs derived using EuroQoL Index tool. UK population norms (n = 1763) by age group are the following, mean (SD) 45–54: 0.85 (0.25); 55–64: 0.80 (0.26); 65–74: 0.78 (0.26); 75+: 0.73 (0.27) [52].

SF-6D = UVs derived from SF-36 using SF-6D algorithm [53].

TTO = time trade-off utility value.

VFQ-25 = 25-item national eye institute visual function questionnaire.

### Stage-dependent changes in visual functioning and QOL

**Figure 1:**
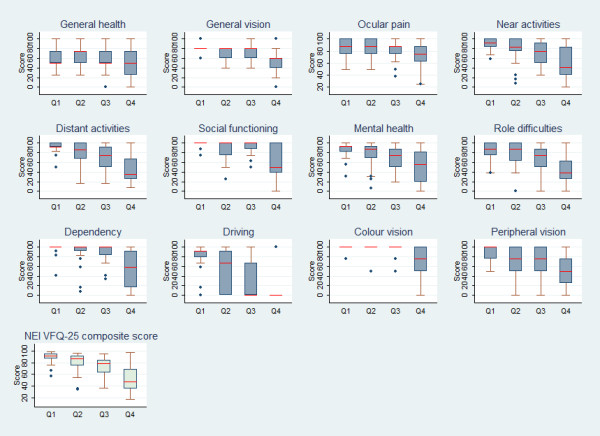
Median (IQR) VFQ-25 composite and subscales scores by IVF quartile.

The VFQ-25 composite score correlated well with IVF (r = −0.67) and VA_B_ (r = −0.71), and its median declined across IVF and VA_B_ quartiles (Figure 1; Kruskal-Wallis P < 0.001). There was strong evidence of a relationship between VFL and every VFQ-25 sub-scale (all P < 0.001), except for general health (P = 0.10) and ocular pain (P = 0.16). The same associations were found for VA_B_ (P < 0.001 for all tests but general health and ocular pain, both P = 0.35). The graphical comparison shows different patterns of change for different dimensions of visual functioning. The driving and peripheral vision sub-scales displayed the largest declines even among patients in the early stages of POAG, with the median score for driving ability equal to 0 in the 3^rd^ and 4^th^ quartiles. Apart from general health and ocular pain, which did not vary, all sub-scales displayed large differences in median scores only in the 4^th^ IVF quartile compared to the others.

Only the SF-36 sub-scales related to its physical component were associated with VFL and VA_B_, (P = 0.03 and P = 0.01, respectively; P = 0.4 and P = 0.92 for the psychological component). No EQ-5D dimension was associated with VFL or VA_B_, except for ‘usual activities’ (P = 0.001).

### Sensitivity of UVs to differences in visual field

Table 3 reports the correlation coefficients between the vision markers and the UVs. The TTO preferences for visual states correlated much better with IVF and VA_B_ than preferences for generic health states. None of the UVs correlated as strongly with vision markers as the VFQ-25 composite score.

**Table 3:** Spearman’s rank correlation coefficients and significance levels between VA, IVF and responses to QOL questionnaires

	**IVF**		**VA**_**B**_	
	***r***	***P-value***	***r***	***P-value***
EQ-5D	−0.25	0.003	−0.19	0.03
SF-6D	−0.29	0.001	−0.22	0.01
TTO	−0.47	<0.001	−0.48	<0.001
VFQ-25	−0.67	<0.001	−0.71	<0.001

IVF = integrated visual field score.

VA_B_ = binocular visual acuity.

EQ-5D = UVs derived using EuroQoL Index tool.

SF-6D = UVs derived from SF-36 using SF-6D algorithm [53].

TTO = time trade-off utility value.

**Figure 2:**
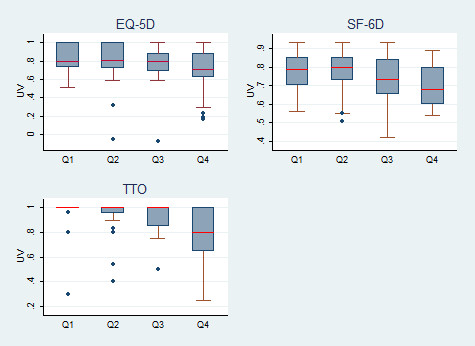
**Median (IQR) of EQ-5D, SF-36 and TTO UVs by IVF quartile.** Q-5D = UVs derived using EuroQoL tool. SF-6D = UVs derived from SF-36 using SF-6D algorithm [53]. TTO = time trade-off utility value.

Figure 2 shows a declining trend in UVs from SF-6D and TTO for increasing VFL, with patients in the 4^th^ quartile showing lower median UVs than those in the first; the significant difference (P = 0.01 and p = 0.001, respectively) was mainly driven by the lower utilities reported by patients in the 4^th^ VFL quartile. Median UVs from EQ-5D did not vary (P = 0.08) nor were significantly different from population norms (Table 2). The comparison of median UVs across VA_B_ quartiles also showed an association with SF-6D (P = 0.02) and TTO (P < 0.001) but not EQ-5D (P = 0.17).

### Sensitivity of UVs to changes in visual functioning

As shown in Table 4, Spearman’s rank correlation coefficients indicate statistical dependence between the VFQ-25 composite score and the UVs from EQ-5D, SF-6D and, in particular, TTO.

**Table 4:** Spearman’s rank correlation coefficients and significance levels between utility values and VFQ-25 scores

**VFQ-25 scores**	**EQ-5D**		**SF-6D**		**TTO**	
	***r***	***P-value***	***r***	***P-value***	***r***	***P-value***
General health	0.56	<0.001	0.49	<0.001	0.12	0.226
General vision	0.27	0.002	0.24	0.004	0.45	<0.001
Ocular pain	0.42	<0.001	0.28	0.001	0.24	0.014
Near-vision activities	0.37	<0.001	0.42	<0.001	0.6	<0.001
Distance-vision activities	0.31	<0.001	0.41	<0.001	0.56	<0.001
Social functioning	0.24	0.007	0.36	<0.001	0.50	<0.001
Mental health	0.37	<0.001	0.44	<0.001	0.57	<0.001
Role difficulties	0.36	<0.001	0.48	<0.001	0.55	<0.001
Dependency	0.41	<0.001	0.48	<0.001	0.57	<0.001
Driving	0.24	0.018	0.21	0.057	0.46	<0.001
Colour perception	0.22	0.015	0.36	<0.001	0.45	<0.001
Peripheral vision	0.26	0.004	0.38	<0.001	0.45	<0.001
***Composite score***	0.38	<0.001	0.43	<0.001	0.60	<0.001

EQ-5D = UVs derived using EuroQoL Index tool.

SF-6D = UVs derived from SF-36 using SF-6D algorithm [53].

TTO = time trade-off utility value.

VFQ-25 = 25-item national eye institute visual function questionnaire.

All the individual VFQ-25 dimensions correlated well with TTO (r ≥ 0.50 and P < 0.001 for all tests) except for general health and ocular pain. Correlations were instead weak between UVs from EQ-5D and SF-6D and all sub-scales other than general health. In fact, TTO was sensitive to changes in the largest number of vision-specific sub-scales, capturing up to 25% more of the impact of vision loss on functionality dimensions such as driving and social functioning (in terms of the difference between correlation coefficients) compared to EQ-5D and SF-6D.

## Discussion

Although official guidelines on QALY estimates recommend generic health-state valuations that reflect the preferences of the general population to improve the comparability and generalisability of results, such valuations may not detect changes in health status for some conditions [40]. The low sensitivity of generic instruments can be attributed to the irrelevance of some of their questions to vision-related QOL. For instance, EQ-5D asks about problems with mobility but these may not occur until the very last stages of POAG, if at all. Moreover, the three response options might not detect QOL changes of a smaller magnitude. To address issues of poor sensitivity and ceiling effects displayed by the original instrument, a 5-level version of EQ-5D was recently introduced, thus potentially improving its performance at the milder end of visual function loss [22,41]. Similar conclusions can be drawn about the performance of SF-6D: it detects a minimally important difference in QOL, perceived by patients as beneficial, that is different in absolute terms but proportionally equivalent to that of EQ-5D [42]. Genuine differences of a smaller magnitude are therefore undetected by both instruments. Lack of specificity may compound the problem as, for instance, generic measures may not reflect glaucoma patients’ preference concerning both disease symptoms and avoidance of treatment side-effects. Our findings on EQ-5D are in line with existing literature showing no significant association between EQ-5D scales and glaucoma severity [19,43]. Results in other chronic conditions, ophthalmic and non- (including macular degeneration, rheumatoid arthritis and asthma), indicate that EQ-5D has limited disease-specific sensitivity [44-46]. A complementary explanation has to do with the fact that the frequency and intensity of negative thoughts has an impact on responses to QOL questionnaires [47]. In this sense, since generic tools do not require patients to focus on the state of their vision, existing problems with vision-related functioning might not be captured in their score.

The higher sensitivity to glaucoma-induced QOL changes of the TTO adapted to vision is consistent with the question being specific to vision, and has also been documented by Aspinall et al. (2008), who observed declining TTO utilities with increasing severity while EQ-5D remained unaffected [8]. However, TTO has high ceiling effects and does not decrease with deteriorating vision and visual functioning in the earlier stages of disease (median UV = 1 for first 3 quartiles): preferences may indeed not change until end-stage POAG, as patients adapt to gradual peripheral vision loss, or the elicitation methods may be inadequate. Ceiling effects for generic utility scales are widely reported for ophthalmic conditions and in general population surveys [48,49]. The high documented percentage of zero-traders with TTO (UV = 1) may be explained with a ‘threshold of tolerability’ that should be reached before patients are willing to sacrifice even a few days [50]. Similar findings with Standard Gamble utilities confirm that glaucoma patients are willing to accept lower risks in return for perfect health (UVs closer to 1) than patients with other eye conditions such as refractive error or diabetic retinopathy [51]. For the same patients, utilities did decline once the anchor points were shifted from death and perfect health to blindness and perfect vision but the discrepancy between the two sets of UVs remained largest with increasing disease severity [51]. EQ-5D was also appears to lack sensitivity to mild conditions and small changes in health status [4].

Willingness to trade time was found predominantly among patients with severe glaucoma, characterised by poorer central VA; however, central VA might not deteriorate until later stages of POAG, leaving VFL as the main determinant of early QOL changes. VFL affects a selected number of functionality dimensions that might not be encompassed within generic utility elicitation methods: some of the reported associations between functional ability and VFL were weaker than those with VA [11]. Here, instead, all the associations found for VA_B_ and IVF were matched, thus confirming the relevance of these findings for glaucoma patients, whose vision loss is mainly peripheral. In line with existing literature, we found that the VFQ-25 subscales showing the lowest scores in POAG are driving, peripheral vision and activities involving both near and distant vision [20,21].

Our study has some limitations. Firstly, there are significant differences in the TTO, EQ-5D and SF-6D methods which limit their comparability. The TTO does not value health states worse than death, and is anchored differently (perfect vision/death) to the EQ-5D and SF-6D (perfect health/death). Since the implementation of this study, a debate has arisen as to whether utilities anchored at perfect vision/death are measuring the same construct as those anchored at perfect health/death, and therefore whether they are appropriate for calculating QALYs used in cost-utility analyses [51]. Secondly, the use of non-parametric statistical methods implies that patients’ circumstances, which might influence individual perceptions of sight loss as a disability, were not controlled for. However, univariable analyses showed that none of the socio-demographic and clinical characteristics were likely to confound the observed associations. Furthermore, recruitment from one single London-based hospital potentially limits the generalisability of findings. Finally, comparisons with other studies are hindered by the lack of an agreed system of glaucoma staging.

## Conclusions

This study demonstrates that POAG reduces visual functioning (VFL, VA) and vision-related QOL (VFQ-25 scores), affecting the 3 UVs to different extents: those from TTO were the most sensitive, followed by SF-6D and then EQ-5D, which was mostly unaffected. In general, TTO utilities were highest in value and had the strongest relationship with IVF, VA_B_ (known-group differences) and the VFQ-25 composite score (convergent validity), implying higher overall sensitivity to glaucoma-induced QOL changes.

Although TTO UVs correlated with glaucoma-induced QOL changes better than those from SF-6D and EQ-5D, our results do not go as far as to suggest that TTO’s sensitivity is sufficiently adequate for POAG. In fact, none of the generic instruments analysed appeared very sensitive to differences in VFL or perceived visual function for patients at milder stages of visual impairment. The correlations of TTO UVs with ophthalmic and functionality measures may thus be mainly driven by their drop among patients with end-stage POAG. Preferences may be genuinely unchanged early on, elicitation methods may be inadequate, or both. As several dimensions of visual functioning were demonstrably affected from the earlier stages, these UV elicitation methods are very likely inadequate. This finding is crucial for the timing of treatment against degenerative conditions such as POAG, as cost-utility analysis should employ valid utility measures that can detect genuine changes in preferences over different stages. Underestimation of QALYs gained by patients undergoing interventions to slow POAG progression might lead to delays in starting beneficial therapies, which can cause unnecessary utility losses. Although the use of TTO adapted for vision should be encouraged over other generic QOL tools, economic studies should question the validity of standard measures and identify, develop or validate in practice better methods where necessary.

## Abbreviations

POAG: Primary open-angle glaucoma; VF: Visual field; QALY: Quality-adjusted life years; UV: Utility value; QOL: Quality of life; VFL: Visual field loss; NICE: National institute for clinical excellence; VFQ-25: National Eye Institute 25-item visual function questionnaire; EQ-5D: EuroQoL quality of life scale; TTO: Time trade-off; SF-36: Medical outcomes study 36-item short form; SF-6D: 6-dimensions utility classification system for SF-36; VA_B_: Binocular visual acuity; IVF: Integrated visual field; MEH: Moorfields Eye Hospital; IQR: Inter-quartile range; CI: Confidence interval; ANOVA: Analysis of variance; SD: Standard deviation.

## Competing interests

This study was supported by an Investigator Initiated Research grant from Pfizer Inc., New York Headquarters (2005–0570).

## Authors’ contributions

FB performed the statistical analysis and drafted the manuscript. The study was conceived by YA, who carried out data collection and helped with drafting the manuscript. MJB collaborated with the study design and implementation. HK assisted with data analysis and provided feedback on successive drafts. All authors read and approved the final manuscript.

## Pre-publication history

The pre-publication history for this paper can be accessed here:

http://www.biomedcentral.com/1471-2415/12/43/prepub
